# Multi-modality imaging to determine the cellular heterogeneity of nasopharyngeal carcinoma components

**DOI:** 10.18632/oncotarget.1894

**Published:** 2014-04-11

**Authors:** Weidong Zhang, Yanling Zhang, Shi Ke, Mingjian Lu, Guang Yang, Tao Zhang, Jianjun Han, Zhenyin Liu, Wei Wang, Henry Ran, Chaoxia Zou, Shaofan Hu, Guangtao Lei, Chuanxing Li, Fujun Zhang

**Affiliations:** ^1^ State Key Laboratory of Oncology in South China, Department of Imaging and Interventional Radiology, Cancer Center, Sun Yat-sen University, Guangzhou, Guangdong, 510060, P. R. China; ^2^ School of Biotechnology, Southern Medical University, Tonghe, Guangzhou, Guangdong, 510515, P.R. China; ^3^ Department of Radiology, Baylor College of Medicine, One Baylor Plaza, Houston, Texas, 77030, USA; ^4^ Department of Biochemistry and Molecular Biology, Harbin Medical University, Harbin, Heilongjiang, 150081, P. R. China; ^5^ Jiangxi Children's Hospital, Nanchang, Jiangxi, 330006, P.R. China; ^6^ Department of Cardiology, Second Affiliated Hospital of Nanchang University, Nanchang, Jiangxi, 330006, P.R. China

**Keywords:** CT, FDG, CXCR4, retinoid acid, MMP, molecular imaging, multi-agent imaging, multi-modality imaging, optical imaging, PET

## Abstract

Nasopharyngeal carcinoma (NPC) is an endemic public health problem in South and Southeast Asian countries. The disease components at the molecular level are unclear and need exploration for the development of future individualized molecular medicine. The purpose of this study was to test the feasibility of target-specific agents to detect different components of NPC. The binding capability of human NPC cell lines was determined by incubation with either agents that specifically target the metabolic status, host cytokines, and stroma. Mice bearing human NPC xenografts were injected with the same test agents plus a clinical molecular imaging agent (^18^F-fluorodeoxyglucose) and computer tomography (CT) contrast agent. *In vitro* cell studies have demonstrated that target-specific agents bind to NPC cells with significantly higher signal intensities. Those agents not only bound to the cell membrane but also penetrated into the cytosol and cell nuclei. *In vivo* imaging demonstrated that the human NPC xenografts revealed high glucose uptake and a profound vasculature in the tumor. All agents were bound to the tumor regions with a high tumor-to-muscle ratio. Finally, all imaging data were validated by histopathological results. Multiple, target-specific agents determine the dynamic and heterogeneous components of NPC at the molecular level.

## INTRODUCTION

Nasopharyngeal carcinoma (NPC) is an endemic public health problem in South and Southeast Asian countries [1] with an incidence rate as high as 26.9 per 100,000 persons [2, 3]. Current diagnosis still relies on non-specific imaging modalities such as X-ray, magnetic resonance imaging (MRI), computed tomography (CT) or ^18^F-fluorodeoxyglucose (FDG)/positron emission tomography (PET). Additionally, the therapeutic regimens are standard for all patients. Thus, both the diagnosis and treatment lack specificity to the tumor components based on individual differences. Therefore, most patients only obtain a correct diagnosis at an advanced disease stage [4, 5], and their treatment outcomes are inconsistent. Furthermore, both the diagnostic and therapeutic approaches listed above critically need improvements, considering the individual differences of each patient, different disease types, variable disease stages, and various disease components. In addition, because advanced disease requires a multidisciplinary team to establish an optimal treatment strategy, a correct combination based on each patient's need and disease status is necessary to provide better disease control [6]. Understanding of the disease at the molecular level will improve the accuracy of diagnosis, efficiency of treatment and development of new treatment regimens. A regimen that combines target-specific therapies and is individualized to fit the personal needs of each patient will yield better outcomes and reduce systematic toxicity. Therefore, exploration of molecular targets becomes critical in the field.

It is well known that cancer is an evolving, dynamic, and heterogeneous system; the difficulty with its diagnosis and treatment supports the use of simultaneous, multi-target therapies [7]. Cellular heterogeneity and the timing of expression are critical characteristics of oncogenic events and provide a framework to interpret pathological, diagnostic, and therapeutic observations of tumors [7]. Current molecular imaging technology provides a noninvasive approach to accurately study multiple disease components longitudinally. The combination of nuclear medicine, optical molecular imaging, X-ray, and CT allows simultaneous detection of tumor molecular events.

In the present study, we utilized human NPC cell lines and tumor xenografts in nude mice to determine multiple disease components at the cellular or tumor levels. We found that NPC shows a high metabolic activity, cytokine response and stroma modification. Our data demonstrate that a single tumor has multiple cellular components, including tumor xenografts derived from homogeneous cell populations with the same genetic background and at the same time. We hope this work provides a different view of the complexity of human cancer and supports the use of simultaneous, multi-target therapies for treating cancer.

## RESULTS

### *In vitro* imaging

**Fig 1:**
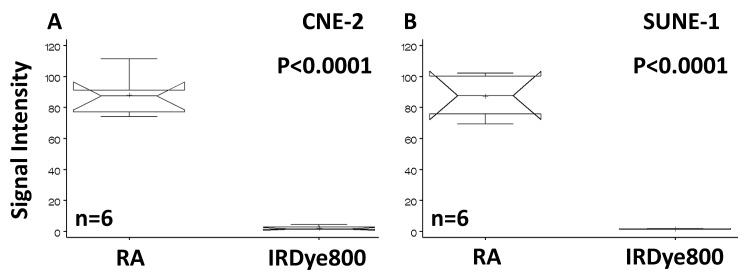
Statistical analysis and comparison of signal intensity of cells binding to RA Human NPC cells CNE-2 (A) and SUNE-1 (B) bound to the RA agent but not to the free reporter. The signal intensities of the RA agent are significantly higher than those of the dye.

Binding of the retinoid acid (RA) agent to the NPC cell lines have been demonstrated in the previous publication [16, 17]. A further detail analysis and comparison of signal intensity demonstrated that the signal intensity of RA agent is statistically higher than that of the free dye (Fig. 1) with p-value of less than 0.0001.

**Fig 2:**
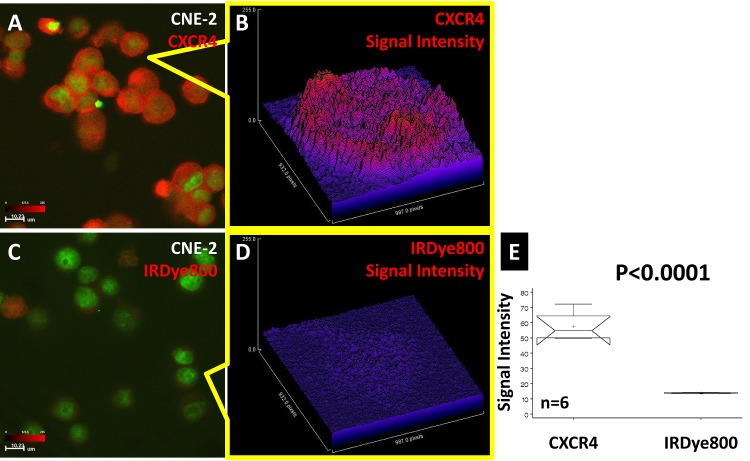
CXCR4 confocal imaging of human NPC CNE-2 cells The CXCR4 agent bound to CNE-2 cells (A). Single-cell signal intensity analysis showed the location of CXCR4 to be inside the cell (B). The corresponding optical dye showed a very weak signal in both the population image (C) and single-cell image (D). Statistical comparison showed that the signal intensity of CXCR4 was significantly higher than that of the free dye (E).

NPC cell line CNE-2 binding to the CXCR4 agent is shown in Fig. 2. This cell line was strongly positive for CXCR4 (Fig. 2A) with weak, nonspecific binding to the reporter dye (Fig. 2C). The signal intensity plots showed that the origins of the signal at the single-cell level had higher signal intensity than that of the cytosol for CXCR4 (Fig. 2B). The signal was diffuse in the cells binding to the nonspecific dye (Fig. 2D). Statistical comparison showed that the signal intensity of CXCR4 was significantly higher than that of the free dye (Fig. 2E).

**Fig 3:**
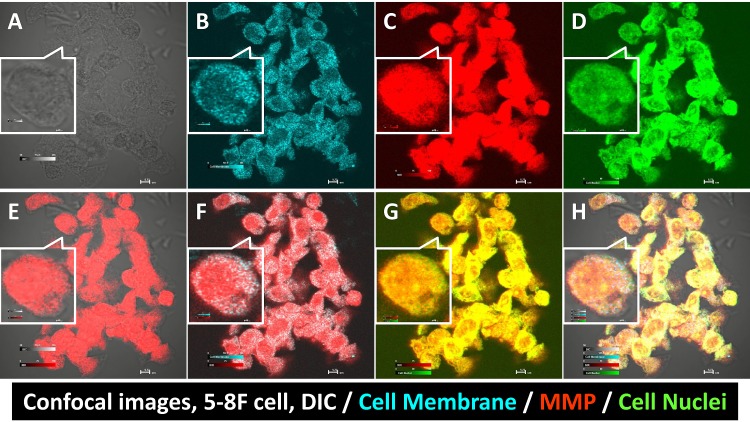
Confocal images of human NPC 5-8F cells binding to the MMP agent Differential interference contrast (DIC) imaging showed the cell edges and morphology (A). CellTracker™ stained the cell membranes (B). MMP bound to multiple cell components (C). Cell nuclear stain verified the cell integrity (D). Merged DIC and MMP imaging showed the MMP agent correlated to the cell morphology (E). MMP was not only present in the cell membrane (F) but also in cell nuclei (G). Merged quadruple signals showed the location of each cell compartment (H).

The NPC cell line 5-8F was studied in detail regarding the binding location of the matrix metalloproteinase (MMP) agent, and the results are shown in Fig. 3. The signal channel images showed the cell morphology (Fig. 3A), cell membrane (Fig. 3B), MMP (Fig. 3C) and cell nuclei (Fig. 3D). Merged morphology and MMP imaging showed that the MMP signal arose from inside the cells (Fig. 3E), a finding that was confirmed by overlaying the cell membrane and MMP images (Fig. 3F). Merged cell nuclei and MMP images demonstrated that the MMP agent also penetrated the cell nuclear membrane (Fig. 3G). Finally, the location and relation of each component are shown in Fig. 3H.

### *In vivo* imaging

**Fig 4:**
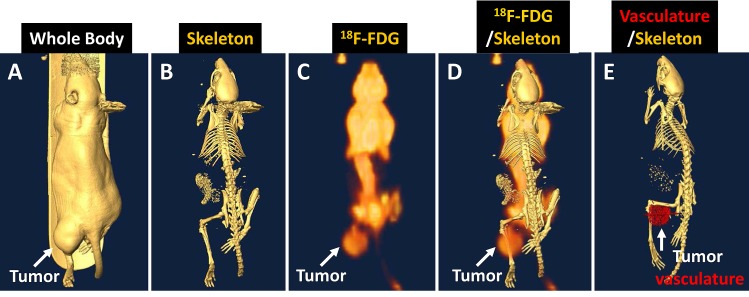
Human NPC tumor-bearing animal images showing the tumor location (A), soft-tissue property without bone damage (B), glucose uptake and distribution in the whole body (C). PET/CT showed high glucose signal intensity in the tumor region (D). CT contrast agent analysis revealed the hypervasculature in the tumor region (E).

Table 1 lists injected molecular agents. Tumor-bearing animals underwent common clinical imaging modalities, and the results are shown in Fig. 4. The CT body image showed the tumor location (Fig. 4A), and the CT skeleton image demonstrated that the tumor mass was a soft tissue tumor without bone damage at that stage (Fig. 4B). The ^18^F-FDG/PET image revealed that the tumor region has a higher glucose uptake than the opposite normal region (Fig. 4C). The merged PET/CT showed the ^18^F-FDG signal intensity at the whole-body level (Fig. 4D). Analysis of the CT contrast agent intensity revealed the high vasculature structure in the tumor region (Fig. 4E).

**Table 1:** Summary of molecular probes in the in vivo imaging study

Target	Agent	Reporter	Injected Dose
Glucose	18F-FDG	18F 511 keV	100 μCi
Vasculature	Omnipaque	Iodine	0.2 ml
Retinoid receptor	Retinoid acid	IRDyeCW 780/830 nm (ex/em)	10 nM
Cytokine	CXCR4	IRDyeCW 780/830 nm (ex/em)	5 nM
Stoma	Matrix metalloproteinase	IRDyeCW 780/830 nm (ex/em)	2 nM

**Fig 5:**
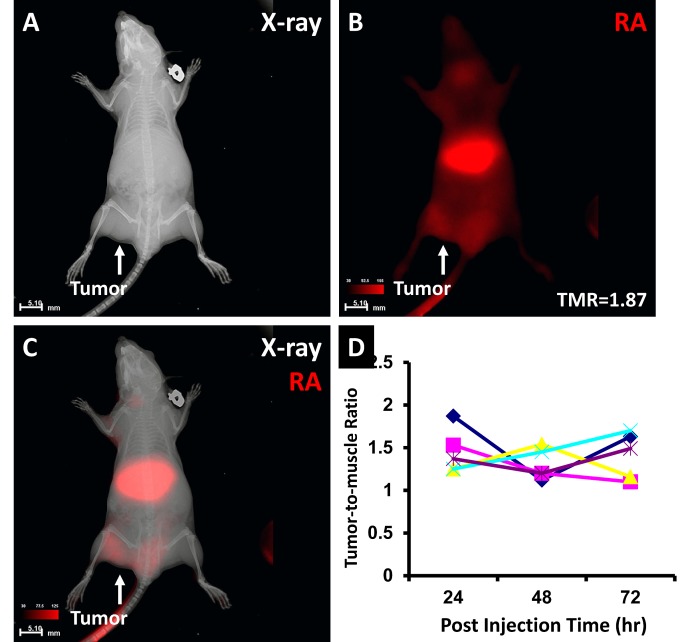
The RA agent binding to the human NPC xenograft X-ray imaging showed the tumor location (A). Optical imaging showed the tumor region with a high RA signal intensity and a TMR of 1.87 (B). Merged X-ray and RA imaging showed the agent in the liver and tumor regions (C). TMR changes in each animal over 72 hour post injection demonstrated individual and dynamic variations (D).

RA binding to the NPC tumor is shown in Fig. 5. X-ray imaging shows the location of the tumor mass (Fig. 5A). Optical imaging shows high RA agent signal intensity in the tumor region with a tumor-to-muscle ratio (TMR) of 1.87. Merged X-ray and optical imaging confirmed that the signal originated from the tumor region (Fig. 5C). The TMR over post-injection time plot demonstrated the variability of each animal at different imaging times (Fig. 5D). No animal showed a consistent TMR during 72 hours.

**Fig 6:**
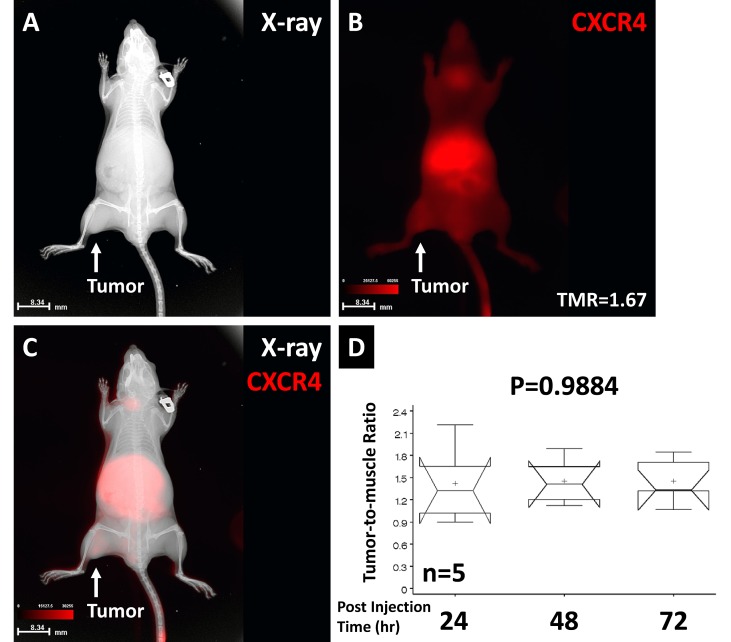
The CXCR4 agent binding to the NPC tumor X-ray imaging showed the tumor location (A). The CXCR4 signal showed whole-body distribution and a high TMR in the tumor region (B). Overlaid anatomic and optical images showed the location of the CXCR4 signals (C). Comparison of TMRs over the 72-hour post injection period showed no significant difference. The high variation at the early time point suggested that the imaging agent required time to be detected in the tumor region (D).

CXCR4 binding to the NPC tumor is shown in Fig. 6. The location of the tumor xenograft is shown by X-ray imaging (Fig. 6A). The whole-body CXCR4 agent distribution is shown by optical imaging (Fig. 6B). The tumor region has a high TMR of 1.67 at 24 h post injection. Merged X-ray and optical imaging demonstrated the anatomic structure and molecular imaging result (Fig. 6C). Comparison of the TMR results over 3 days post injection is shown in Fig. 6E. No statistical TMR difference was found over the 3-day period (P=0.9884).

**Fig 7:**
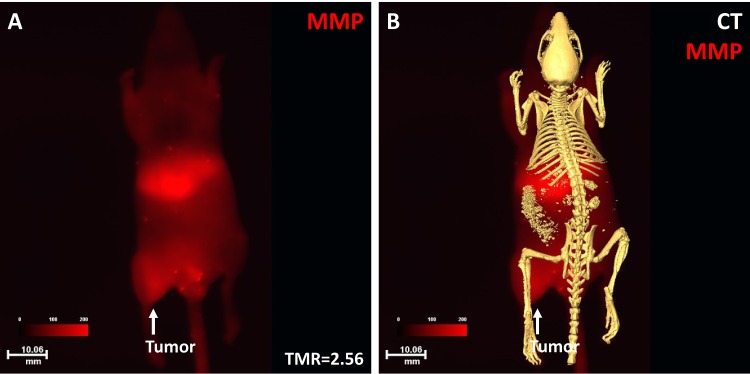
Optical and CT imaging of MMP binding to the NPC tumor Optical imaging showed that the NPC tumor had high signal intensity with a TMR of 2.56 (A). The merged images showed the anatomic location of the tumor and MMP signal distribution at the whole-body level (B).

The MMP agent binding to the NPC tumor is shown in Fig. 7. Optical imaging showed high MMP signal intensity in the tumor region with a TMR of 2.56 (Fig. 7A). Merged optical and CT skeleton imaging demonstrated the nature of the soft-tissue tumor without bone destruction at this stage (Fig. 7B).

**Fig 8:**
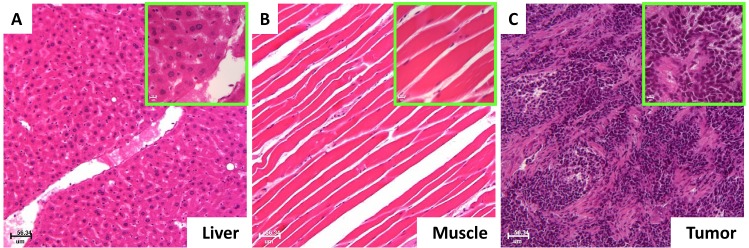
Histopathological validation of the imaging results Histological results showed that the normal organ with the high signal intensity was the liver (A) and the organ with the low signal intensity was the muscle (B). The disease site with the high vasculature, high glucose, RA, CXCR4 and MMP signals represented the tumor (C).

All imaging data were verified by dissected organ imaging (data not shown) and histopathological analysis, and the results are shown in Fig. 8. Both low- and high-magnification imaging validated the liver (Fig. 8A), muscle (Fig. 8B), and tumor (Fig. 8C).

## DISCUSSION

NPC is an endemic public health problem in certain countries with an incidence rate as high as 26.9 per 100,000 persons [1-3]. It is well known that cancer is a complex, dynamic, heterogeneous systematic network of interrelations that vary in different cells, between cells, and at different times in any given cell [7]. Therefore, developing multi-target diagnostic and therapeutic approaches becomes critical to solve this dynamic and spatially heterogeneous problem. Our previous data demonstrate that two different tumor cell lines can develop very different growth patterns in the tumor xenograft. Furthermore, the same tumor xenograft can change the growth pattern and respond to the imaging agent at different time points [14]. In addition, the same cell line in animals with the same genetic background can demonstrate different growth patterns and response to the injected agent even under near-ideal experimental conditions [19]. Together, the above results demonstrate that cancer is a complex, dynamic, heterogeneous disease and support the notion of simultaneous, multi-target diagnoses and therapies for detecting and treating cancer [7].

In the present study, we use NPC as a model to further dissect cancer heterogeneity at the cellular and molecular levels using target-specific molecular imaging. As we have demonstrated in the current study, NPC tumors can be detected by multiple agents from the cellular to whole animal levels. However, unexpectedly, our well-characterized RGD agent [13, 14, 18-24] did not bind to any of the cell lines used in the *in vitro* studies. The latter result prompted us to investigate the tumor vasculature in the *in vivo* model using a CT contrast agent. The CT imaging data showed that NPC contains high volume of blood vessels (Fig. 4E). By contrast, our RGD agent still did not show increased signal intensity in the tumor region (data not shown). We conclude that the binding motif of the NPC tumor may be different than that of the other tumor vessels that we tested previously, and the RGD agent cannot be used as a general agent for detecting this tumor.

Given that human NPC is comprised of a mixture of multiple cells, it is logical to use multiple agents in the present study because of the complementary effects of each agent [19]. The simultaneous injection of multiple agents is expected to increase the positive detection rate. Our data demonstrated that RA, CXCR4 and MMP agents can be used both *in vitro* and *in vivo* to detect NPC. ^18^F-FGD/PET is the most commonly used molecular imaging modality in current clinical practice. However, our imaging data showed its limitation in NPC detection before the tumor metastasizes to other locations. The high glucose uptake in the normal brain and neck regions results in a low TMR and makes it difficult to distinguish the tumor from normal tissue (Fig. 4 C and D) due to the limitation of spatial resolution.

The dynamic change in each tumor is clearly shown in Fig. 5D. Despite the increased TMR during the 72-hours post injection period, each animal shows a different TMR at each imaging time. The data demonstrate that the image only represents one time point result of dynamic interaction between the tumor and imaging agent. Furthermore, one cannot make conclusions about disease status based solely on one imaging time point with one imaging modality. Although some agents can be imaged over a long period of time without statistical difference (Fig. 6D), the variances of TMR over time re-emphasize the needs for dynamic imaging to detect dynamic tumor change.

*In vivo* imaging techniques have several advantages in pre-clinical researches. The combination of genetic modified the cell lines with optical reporters and injectable target-specific molecular agents let us simultaneously study tumor growth, metastasis pattern and marker expressions. The high spatial resolution of optical imaging provides the advantage of imaging tumor characteristics from whole body to cellular levels [14, 25].

Finally, the results concerning our agents that penetrate into cells suggest that they can be used as carriers for therapeutic purposes. A chemotherapeutic agent can be attached to a targeting component, changing the molecule into a target-specific chemotherapeutic agent. Because the original chemistry design is already available for this purpose, we can easily obtain three target-specific agents for treatment according to tumor marker expression status. The drawbacks of this strategy are the tissue distribution of the agent and potential organ-specific toxicity. As images have already demonstrated that all the agents show high uptake in the liver, it is obvious that the liver toxicity will more likely occur when using any of these agents at a high dose. Therefore, balancing the therapeutic efficacy and normal organ toxicity relies on capturing the therapeutic window at the correct dynamic time point for the tumor [14, 16, 18, 19]. We believe noninvasive, target-specific molecular imaging will be a tool to help further understand the dynamic changes and heterogeneity in tumors.

## MATERIALS AND METHODS

### Cell lines

Human cancer cell lines (CNE-2, SUNE-1, 5-8F, and CCL-30) were grown in culture in Dulbecco's Modified Eagle's Medium with high glucose or F12 medium (Life Technologies, Grand Island, NY), supplemented with 10% fetal bovine serum (Hyclone, Logan, UT), in incubators with 5% CO_2_ at 37°C.

### Tumor xenografts

Nude mice (4- to 6-week-old; 18–22 g; Harlan, Indianapolis, IN) were maintained in a pathogen-free mouse colony in a facility accredited by the American Association for Laboratory Animal Care, and all experiments were performed in compliance with the guidelines of the Institutional Animal Care and Use Committee. For tumor implantation, cultured tumor cells were harvested near confluence by treating monolayers with 0.05% trypsin-ethylenedinitrilotetraacetic acid. Cells were pelleted at 130 × *g* for 5 min and resuspended in sterile phosphate-buffered saline. Approximately 1 × 10^6^ cells were implanted subcutaneously into each mouse.

### Imaging agent synthesis

All agents were designed and synthesized in-house as previously described [8-19]. They were purified by high-performance liquid chromatography (HPLC) and confirmed by mass spectrometry, analytic HPLC, and fluorescence spectrophotometry. ^18^F-FDG was purchased from Cyclotope (Houston, TX). The CT contrast agent Omnipaque was purchased from GE Healthcare (Fairfield, CT).

### Confocal microscopic imaging

The human NPC cell lines 5-8F, CCL-30, CNE-2, and SUNE-1 were used for *in vitro* binding analysis. All cells were co-incubated with either the imaging agents or their reporter dye for 30 min at 37°C. Cells were fixed and counterstained with 1 microM Sytox Green (Life Technologies) in 95% ethanol for 15 min at 4°C. For cell membrane staining, cells were incubated with membrane dye (CellTracker™; Life Technologies) for 10 min at 37°C, and then for 20 min at 4°C.

Stained cells were transferred to slides for microscopic examination. Images were captured using an Olympus confocal microscope (Fluoview 1000; Olympus America, Center Valley, PA). Near-infrared (NIR) dyes were measured at excitation/emission (Ex/Em) wavelengths of 765/810 nm, the cell membrane was examined at 553/570, and cell nuclei were examined at 488/510 nm. Signal intensities were recorded from one slice of multiple z-stacks with 0.5-micrometer gaps. Sytox Green, membrane imaging agent or NIR dye signals were pseudocolor green (Em 510 nm), Cyan (Em 570 nm) or red (Em 810 nm), respectively.

### Animal imaging

Tumors developed after 3 to 4 weeks of growth in the implanted mice to 8–15 mm in diameter. Imaging agents (2–10 nM) were injected into the tail vein of anesthetized mice. Mice were imaged immediately after injection and for as long as 72 hours subsequently. Optical and X-ray images were recorded using the Bruker In-Vivo Multispectral System FX Pro instrument (Bruker Preclinical Imaging, Billerica, MA). *In vivo* PET/single-photon emission CT (SPECT)/CT imaging was performed using the Siemens MicroCAT II SPECT/CT and Inveon PET instrument (Siemens Medical Solutions, Malvern, PA).

### Statistical analysis

SAS software v9.3 (SAS Institute, Cary, NC) was used to analyze data by one-way analysis of variance or the general linear model. Data comparison was presented in notched box-and-whisker plots. The medians (central lines) of two box-and-whisker plots were considered to be significantly different at the 0.05 level (95% confidence), if the corresponding notches did not overlap.

## CONCLUSION

We report preclinical research regarding the use of multi-target-specific molecular imaging agents to determine NPC heterogeneity disease components at the cellular level. More importantly, our data support cancer as a complex network of interrelations that vary in different cells, between cells, and at different times in any given cell. We should consider cancer as an evolving, dynamic, and heterogeneous system and treat cancer using simultaneous, multi-target therapies [7].

## ABBREVIATIONS

CT: Computed tomography
DIC: Differential interference contrast
Em: Emission
Ex: Excitation
FDG or ^18^F-FDG: Fluorodeoxyglucose
HPLC: High-performance liquid chromatography
MMP: Matrix metalloproteinase
NPC: Nasopharyngeal carcinoma
PBS: Phosphate-buffered saline
PET: Positron emission tomography
RA: Retinoid acid
TMR: Tumor-to-muscle ratio
